# Aesthetic monitoring-based assessment of oncological safety of oncoplastic management of breast cancer: a multi-center research study

**DOI:** 10.1186/s12893-021-01410-0

**Published:** 2021-12-07

**Authors:** Selmy Awad, Ahmed Tarabay, Fahad H. Qahtani, Faisal S. Alsulaimani, Mohamed M. Shaat, Mazin A. Ali, Ahmad H. Khalid, Qasem M. Alharthi, Musab A. ALThomali, Shabab S. Althobaiti, Nadiah G. AlAmri, Abdullah M. Alturkistani, Abdullah A. Alshamrani, Amal A. Ali, Ahmed H. Alsufyani, Fahad M. AlSofyani, Abdullah A. Alghuraybi, Amer M. Alnefaie, Shuruq A. Alharthi, Abdulrahman A. Almalki, Abdulrahman S. Alghamdi, Azzah Alzahrani, Ashraf Yehia, Mohamed Samir Abou Sheishaa

**Affiliations:** 1 General Surgery Department, Mansoura University Hospitals, Mansoura, 35516 Egypt; 2 General Surgery Department, King Faisal Medical Complex, Taif, Saudi Arabia

**Keywords:** Oncoplastic, Breast, Aesthetic, Oncological, Safety

## Abstract

**Background:**

Oncoplastic Breast surgeries (OBS) in breast cancer have evolved to preserve the cancerous breast rather than its amputation to improve postoperative cosmetic results. The lack of evidence to support the oncological safety and benefits of OBS is questionable. In this study, we evaluate various aspects of oncoplastic surgeries with a focused monitoring of aesthetic results and oncological safety.

**Methods:**

This was a multi-center observational study focused on the statistics of data collected from cases who underwent oncoplastic surgeries from the cohort of breast cancer candidates at Mansoura University Hospitals/Egypt and King Faisal Medical Complex/KSA from January 2015 to June 2018. All data were analyzed carefully using SPSS v-26.

**Results:**

Eighty cases who underwent different oncoplastic surgeries were included and reviewed for the aesthetic outcome and oncological safety. The recurrence rate was found to be 2.5%. The breast impact treatment scale assessment method was used to analyze the aesthetic outcomes, and average scores were accepted in 90% of patients.

**Conclusions:**

The oncoplastic breast surgeries are feasible and they had a high rate of oncological safety with the maintenance of good aesthetic outcomes and patient satisfaction.

## Background

The mortality rate of breast cancer has decreased by 40% due to new diagnostic and therapeutic modalities. Oncoplastic Breast Surgery (OBS) has evolved to preserve the cancerous breast rather than its amputation by allowing larger tumor resections with minor cosmetic alterations. While breast-conserving surgery (BCS) is appropriate for 60–80 breast cancer patients, still many women will require mastectomy [1, 2].

OBS merges the principles of oncologic and reconstructive surgery addressing tissue defects and optimizing cosmesis from breast cancer surgery with negative histologic margins satisfying the reshaping desires of the patient as it may provide the optimal local control while maintaining a cosmetically acceptable breast. A form of breast-conservation surgery, it includes oncologic resection with a partial mastectomy, ipsilateral reconstruction with volume displacement, or replacement techniques with possible contralateral symmetry surgery, as appropriate [3, 4].

Advances in the neoadjuvant chemotherapy and plastic techniques with breast reconstruction changed the field dramatically and became an inaugural part of the art of breast surgery as they improve the patient’s self-esteem without long hospital stay by eliminating the need for any further corrections of surgical defects that result from breast cancer therapies [5–7]. The OBS technique is defined as level 1 and level 2. In level 1, it is used to prevent tumor deformities that are less than 20% of total breast volume. In level 2, the volume loss for tumor excision is larger than level 1 involving the reshaping of breast parenchyma along with reduction of the skin envelope [4].

The involved resection margins are an important factor associated with local recurrence after OBS which are found in 20–40% of all the standard BCS [8–10]. Wider resections with OBS could reduce the positive margin rate and the need for re-operations in comparison to standard BCS. This advocates BCS as re-excision would not only delay the adjuvant treatment and compromise the cosmetic outcome but also creates extra stress in patients as well as a family along with the increased cost of treatment [11–14].

Changes in the physical appearance of females after breast surgery have gained a lot of attention from psychosocial oncologists. Chronic distress observed in one of three females with breast cancer hampered the resumption of their normal life after cancer [15, 16]. The OBS may reduce BID in women undergoing surgery However, cosmetic outcomes of OBS have not been explored much, and only limited studies have evaluated this aspect by a validated aspect with promising good outcomes [17–20]. The lack of evidence to support the oncological safety and aesthetic benefits of OBS is questionable and it led us to work on this aspect. The purpose of this study was to investigate women who had undergone OBS as regards focused monitoring of their aesthetic results using BITS in conjunction with the assessment of oncological safety.

## Methods

### Study design and recruitment

This was a multi-center prospective observational study concerned with the statistics of the data collected for all populations who underwent OBS for breast cancer from the cohort of breast cancer patients at Mansoura University Hospitals/Egypt and King Faisal Medical Complex/KSA during the period of January 2015 to June 2018. This study was conducted on 80 women with breast cancer. The malignant mass range in the included women was from 1 to 5 cm. All the patients had completed the postoperative careful evaluation, including clinical and radiological examination.

Ethical approval was secured from the local Institutional Research Board of Mansoura Faculty of Medicine before the start of the study with code No. MD/136 in October 2014. Written consent had been taken from all participants for participation in the study.

### Inclusion and exclusion criteria

All cases of OBS were included with no age limits including those with preexisting aesthetic concerns like macromastia or significant ptosis that was addressed at the time of cancer resection.

Cases of advanced cancer, deformed breast, refusal of reconstruction, and unilateral reconstruction of bilateral breast cancer were excluded.

### Preoperative workup

The recruited cases were evaluated by Triple Assessment test via:

I—Physical evaluation, II—Mammography ± US.

III—Pathological evaluation: A-Fine Needle Aspiration (FNA); B-Core needle biopsy.

All cases had complete routine laboratory tests pre-operatively. The cases signed an informed consent form for five different types of OBS.

### Discrepancy of the OBS technique

Different types of operative techniques were performed based on the clinical parameters of the patient considering Care coordination with a plastic surgeon interested in immediate breast reconstruction at the time of optimal local control including a partial mastectomy. As an oncoplastic surgical team including the plastic surgeons, we worked together in designing mastectomy skin incision patterns and OBS techniques.

The choice of oncoplastic surgical technique is based upon multiple factors including the Excision volume and breast density (the main parameter of the discrepancy of the OBS surgical technique as shown in Table 4), the location of the cancer, the degree of anatomic ptosis, the desires of the patient, the patient's overall health, and the skill of the surgeon.

These included lateral segmentectomy (LS) and nipple-areola complex (NAC) transfer NAC with sentinel lymph node biopsy (SLNB) with/or without axillary clearance (AC), nipple-sparing mastectomy (NSM) with AC, and immediate latissimus dorsi myocutaneous flap (ILDMF), skin-sparing mastectomy (SSM) with AC and ILDMF, and therapeutic reduction mammoplasty with AC.

### Histopathological analysis and other parameters recorded

Various parameters like breast side where the cancer was located, location as per quadrant were recorded. Histopathological analysis was done to evaluate the histological type of breast carcinoma. The presence of various breast cancer-specific receptors such as estrogen receptors (ER), progesterone receptors (PR), and HER2-new (c-erb2) was also studied. The analysis of the stage of tumor was done by histopathology.

### Analysis of CEA and CA15-3

The serum concentration of CEA was determined using an Enzyme Immunoassay kit, and serum CA15-3 levels were determined using a radioimmunoassay kit. The threshold levels of 0–5 ng/ml CEA and 0–25 U/ml CA15-3 were considered as ‘normal’ levels of the respective markers. Levels > 5 ng/ml CEA or > 25 U/ml CA15-3 were considered elevated in patients.

### Recurrence

Another major outcome analyzed was the local recurrence of breast cancer.

### Assessment of the aesthetic results using BITS [16]

*The time-setting of aesthetic score obtaining*; All patients had a careful postoperative aesthetic evaluation as follows: The 1st year: once every 3 months, and 2nd year: once every 6 months. The aesthetic score assessment was initiated after finishing the course of radiotherapy within the first 3 months. During each follow-up setting for evaluation of aesthetic outcome, the patients were evaluated by BITS, which was derived from previous breast cancer research evaluating aesthetic concerns of women post-operatively.

It assesses the intrusive and avoidant response to the hypothesized traumatic event of surgical treatment of breast cancer (cognitive aspect). Intrusive response questions evaluate pervasive thoughts as “things i see or hear remind me that my body is different”. Avoidant response questions measured limited cognitive experience, subjective awareness of emotions surrounding the event, as “I feel self-conscious about letting my partner see my scar”. It is a questionnaire of 15 items, and each one is given 4 points on the scale (0 = not at all, 1 = rarely, 3 = sometimes, and 5 = often). The sums of overall items range from 0 to 75, which indicates the severity of BID: 0–25 = mild, 26–43 = moderate, and 44+ = severe.

The BITS questionnaire includes the following 15 points;

1. I feel uncomfortable about being seen naked.
2. I avoid looking at and/or touching my scar.
3. I am bothered by feeling or thoughts of bodily disfigurement.
4. I feel self-conscious about letting my partner (person with whom I am sexually intimate) see my scar. Even if you do not have a partner now, rate how you believe you would feel.
5. When i see other women, i think my body appears different than theirs.
6. I have waves of strong feeling about the way my body looks.
7. I think about how my body looked before I was treated.
8. I am reminded of my scar when I pick out clothes to wear.
9. Things i see or hear remind me that my body is different now.
10. I avoid letting myself get emotional when I think of how my body has changed.
11. How my body has changed pops into my mind.
12. I don’t want to deal with how my body looks.
13. I try not to think about my breasts being different.
14. I think about how my treatments my affect my sexual life.
15. I turn away when I must undress in front of my partner (person with whom i am sexually intimate) see my scar. Even if you do not have a partner now, rate how you believe you would feel.

### Data analysis and statistical analysis

All data of the study population were tabulated and analyzed carefully by using SPSS v-26 (IBM, Armonk, NY) for cosmetic outcomes in conjunction with oncological safety. Categorical variables were presented as proportions. Continuous variables were presented as means when symmetrical or medians and ranges when asymmetrical. The t-test was used for the comparison between groups. The counting data were statistically described by frequency or constituent ratio. Chi-square test or Fisher’s exact test was used for comparison between the groups. P-value < 0.05 was considered significant.

## Results

### Demographic data, clinical characteristics, and surgical data for patients

The recruited population included 80 women aged 25–71 years (44.5 + 10.3), who had malignant swellings with sizes of 1–5 cm; 48 cases were of the left breast (60%), and 32 cases were of the right breast (40%). A total of 66 masses were located at the upper outer quadrant (82.5%), 10 were located at the lower outer one (12.5%), and two masses in each quadrant of the upper and lower inner quadrants.

Twenty patients had palpable mobile unilateral lymph nodes (25%), while impalpable status was observed in 60 patients (75%). The other side was free in 76 patients (95%), and benign lesions were detected in 4 patients (5%).

CEA was raised up preoperatively in 62 patients (77.5%) and was average in 18 (22.5%). CA15-3 had an elevation of values in 44 patients (55%) and within normal limits in 36 patients (45%). Several oncoplastic surgeries done for different patients are demonstrated in Table 1.

**Table 1:** Oncoplastic breast surgeries done for different patients

Operation	n	%
LS + NAC transfer + AC	48	60
LS + NAC transfer + SLNB	4	5
Reduction mammoplasty + AC	4	5
SSM + AC + ILDMF	10	12.5
NSM + AC + ILDMF	14	17.5

Lateral segmentectomy (LS); nipple-areola complex (NAC); sentinel lymph node biopsy (SLNB); axillary clearance (AC); nipple-sparing mastectomy (NSM); immediate latissimus dorsi myocutaneous flap (ILDMF); skin-sparing mastectomy (SSM)

### Anatomo-histo-pathology results

Invasive duct carcinoma was discovered in 78 patients (97.5%) of post-operative pathology, and infiltrating lobular carcinoma was observed in 2 patients (2.5%). Only 4 patients (5%) had intra-ductal situ components.

ER, PR, and HER2-new (c-erb2) were positive in 56 patients (70%), 52 patients (65%) and 24 patients (30%), respectively.

The tumor stages were as follows: 72 patients (90%) were stage I, 6 patients were stage II (7.5%), and 2 patients were stage III (2.5%).

All the study population had a contrast enhanced pelvi-abdominal, chest CT, and/or PET scan which showed no distant metastases. Oncological safety was guaranteed by regular follow-up and investigation. Clinical evaluations were done for the study population postoperatively at 3, 6, 9, 12, 18, 24, 36, and 48 months with at least 24 months follow-up for the last cases of the study. Only 2 recurrences were detected clinically (2.5%).

All the study population had mammography ± US, MRI (if needed), contrast enhanced pelvi-abdominal and chest CT, and tumor markers (CA15-3 and CEA) once/year. The results indicated that 97.5% of patients were free from cancer, and only 2 cases showed local recurrence, which was confirmed radiologically. These 2 recurrent cases (2.5%) had tumor markers normal at the first 20 months and then showed elevated markers.

### Data of the aesthetic outcomes

For each patient, BITS had been performed and recorded during each follow-up visit. The average aesthetic scores for each patient of the 6 visits were taken and are shown in Table 2.

**Table 2:** The results of time-related BITS scores and average aesthetic scores

n	BITS (3 m)	BITS (6 m)	BITS (9 m)	BITS (12 m)	BITS (18 m)	BITS (24 m)	Aver. BITS score	n	BITS (3 m)	BITS (6 m)	BITS (9 m)	BITS (12 m)	BITS (18 m)	BITS (24 m)	Aver. BITS score
1	21	19	19	19	19	17	19.00	41	31	29	26	24	21	19	25.00
2	51	41	41	41	43	41	43.00	42	27	25	24	22	21	19	23.00
3	25	21	21	23	25	21	23.00	43	43	41	41	39	39	37	40.00
4	29	23	23	23	25	21	24.00	44	53	51	50	48	47	45	49.00
5	55	45	45	45	47	45	47.00	45	31	28	26	24	22	19	25.00
6	21	19	19	21	21	19	20.00	46	25	24	23	21	20	19	22.00
7	23	21	19	19	17	15	19.00	47	21	20	19	19	18	17	19.00
8	25	19	21	21	21	19	21.00	48	22	20	21	17	18	16	19.00
9	42	40	38	39	39	36	39.00	49	21	20	18	20	18	17	19.00
10	23	17	17	17	21	17	19.00	50	26	25	24	22	21	20	23.00
11	29	23	25	25	25	23	25.00	51	47	46	45	45	44	43	45.00
12	25	21	21	23	25	21	23.00	52	50	48	46	44	42	40	45.00
13	47	37	39	39	41	37	40.00	53	23	21	20	18	17	15	19.00
14	57	47	47	47	49	47	49.00	54	23	22	16	18	18	17	19.00
15	29	23	25	25	25	23	25.00	55	23	22	18	19	19	19	20.00
16	23	21	21	23	23	21	22.00	56	21	20	19	19	18	17	19.00
17	23	17	17	17	21	17	19.00	57	22	21	20	18	17	16	19.00
18	21	17	17	19	21	17	19.00	58	23	21	22	20	21	19	21.00
19	23	20	18	19	19	15	19.00	59	26	24	24	22	22	20	23.00
20	25	21	21	23	25	21	23.00	60	23	21	21	22	20	19	21.00
21	23	17	17	17	23	17	45.00	61	24	22	20	22	20	18	21.00
22	23	17	17	17	23	17	45.00	62	25	22	20	20	22	17	21.00
23	19	19	19	19	19	19	19.00	63	26	24	23	24	23	20	23.00
24	19	19	19	19	19	19	19.00	64	27	24	22	24	22	19	23.00
25	21	19	19	21	21	19	20.00	65	23	21	19	21	19	17	20.00
26	23	17	17	19	21	17	19.00	66	19	18	17	17	16	15	17.00
27	21	19	19	19	19	17	19.00	67	18	17	16	17	18	16	17.00
28	25	19	21	21	21	19	21.00	68	22	20	17	18	21	17	19.00
29	27	21	21	23	23	21	23.00	69	27	26	24	26	24	23	25.00
30	25	19	21	21	21	19	21.00	70	44	42	41	42	40	37	41.00
31	23	19	21	21	23	19	21.00	71	22	19	19	18	20	17	19.00
32	25	19	19	19	21	19	21.00	72	46	42	44	45	43	38	43.00
33	27	21	21	23	25	21	23.00	73	28	20	22	22	26	20	23.00
34	27	21	21	23	25	21	23.00	74	27	23	25	23	25	21	24.00
35	23	19	19	19	21	19	20.00	75	50	48	47	48	45	44	47.00
36	25	15	15	15	15	15	17.00	76	23	17	21	19	22	18	20.00
37	23	15	15	17	17	15	17.00	77	21	17	20	18	21	17	19.00
38	23	17	17	19	21	17	19.00	78	23.00	19	20	22	23	19	21.00
39	31	23	23	25	25	23	25.00	79	42	36	41	37	40	38	39.00
40	49	39	39	39	41	39	41.00	80	48	38	40	41	36	43	41.00

**Fig. 1:**
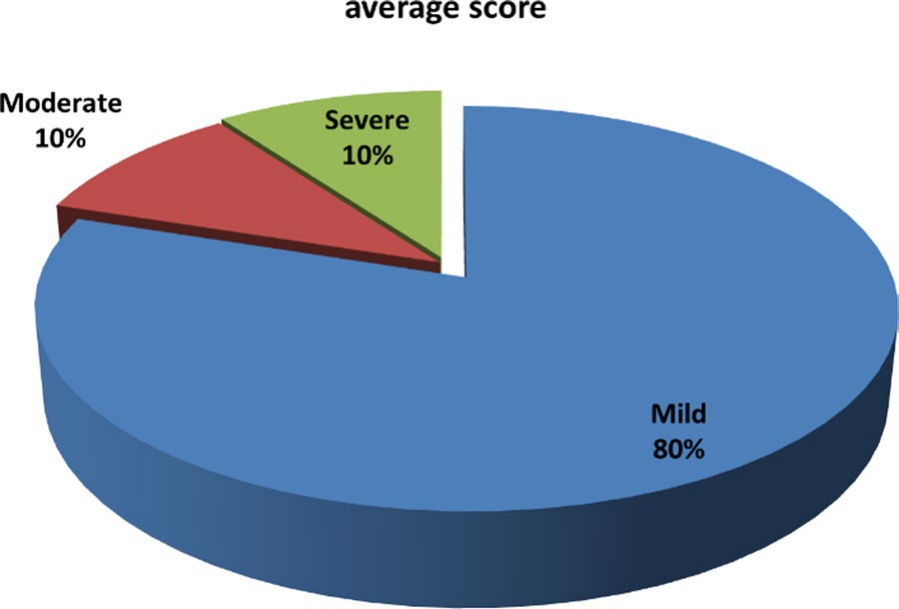
Severity of average scores

The sum of overall items ranges from 0 to 75, which points to the severity of BID as follows: mild (0–25): 64 cases (80%); moderate (26–43): 8 cases (10%); and severe (≥ 44): 8 cases (10%) (Fig. 1). The comparison between post-radiotherapy 3-month BITS and 24-month BITS was reported in Table 3 showing a significant statistical difference (P < 0.05).

**Table 3:** Comparison of post-radiotherapy 3-month BITS and 24-Month BITS

n	BITS (3 m)	BITS (24 m)	P value	n	BITS (3 m)	BITS (24 m)	P value	n	BITS (3 m)	BITS (24 m)	P value	n	BITS (3 m)	BITS (24 m)	P value
1	21	17	0.044	21	23	17	0.039	41	31	19	0.041	61	24	18	0.039
2	51	41	0.027	22	23	17	0.044	42	27	19	0.022	62	25	17	0.044
3	25	21	0.042	23	19	19	0.045	43	43	37	0.050	63	26	20	0.045
4	29	21	0.041	24	19	19	0.039	44	53	45	0.048	64	27	19	0.039
5	55	45	0.022	25	21	19	0.048	45	31	19	0.046	65	23	17	0.048
6	21	19	0.050	26	23	17	0.05	46	25	19	0.049	66	19	15	0.05
7	23	19	0.048	27	21	17	0.048	47	21	17	0.048	67	18	16	0.048
8	25	19	0.046	28	25	19	0.049	48	22	16	0.039	68	22	17	0.049
9	42	39	0.049	29	27	21	0.049	49	21	17	0.044	69	27	23	0.049
10	23	17	0.048	30	25	19	0.049	50	26	20	0.045	70	44	37	0.049
11	29	23	0.039	31	23	19	0.039	51	47	43	0.039	71	22	17	0.049
12	25	21	0.044	32	25	19	0.048	52	50	40	0.044	72	46	38	0.048
13	47	37	0.045	33	27	21	0.05	53	23	15	0.045	73	28	20	0.039
14	57	47	0.039	34	27	21	0.048	54	23	17	0.039	74	27	21	0.044
15	29	23	0.048	35	23	19	0.049	55	23	19	0.048	75	50	44	0.045
16	23	21	0.05	36	25	15	0.039	56	21	17	0.039	76	23	18	0.039
17	23	17	0.048	37	23	15	0.044	57	22	16	0.048	77	21	17	0.048
18	21	17	0.049	38	23	17	0.045	58	23	19	0.05	78	23	19	0.05
19	23	19	0.049	39	31	23	0.039	59	26	20	0.048	79	42	38	0.048
20	25	21	0.049	40	49	39	0.048	60	23	19	0.049	80	48	43	0.049

**Fig. 2:**
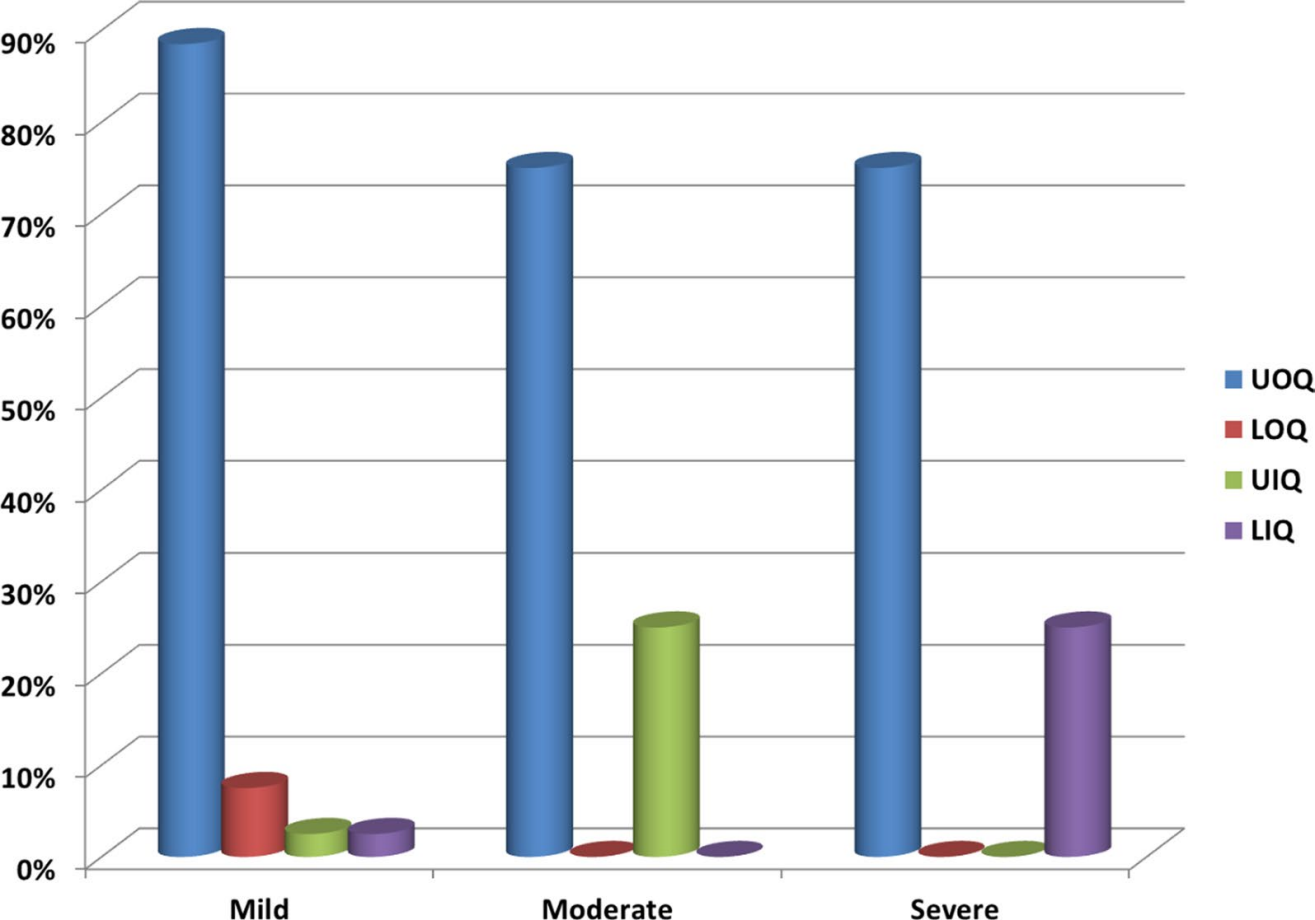
Correlation between average scores and tumor sites

**Fig. 3:**
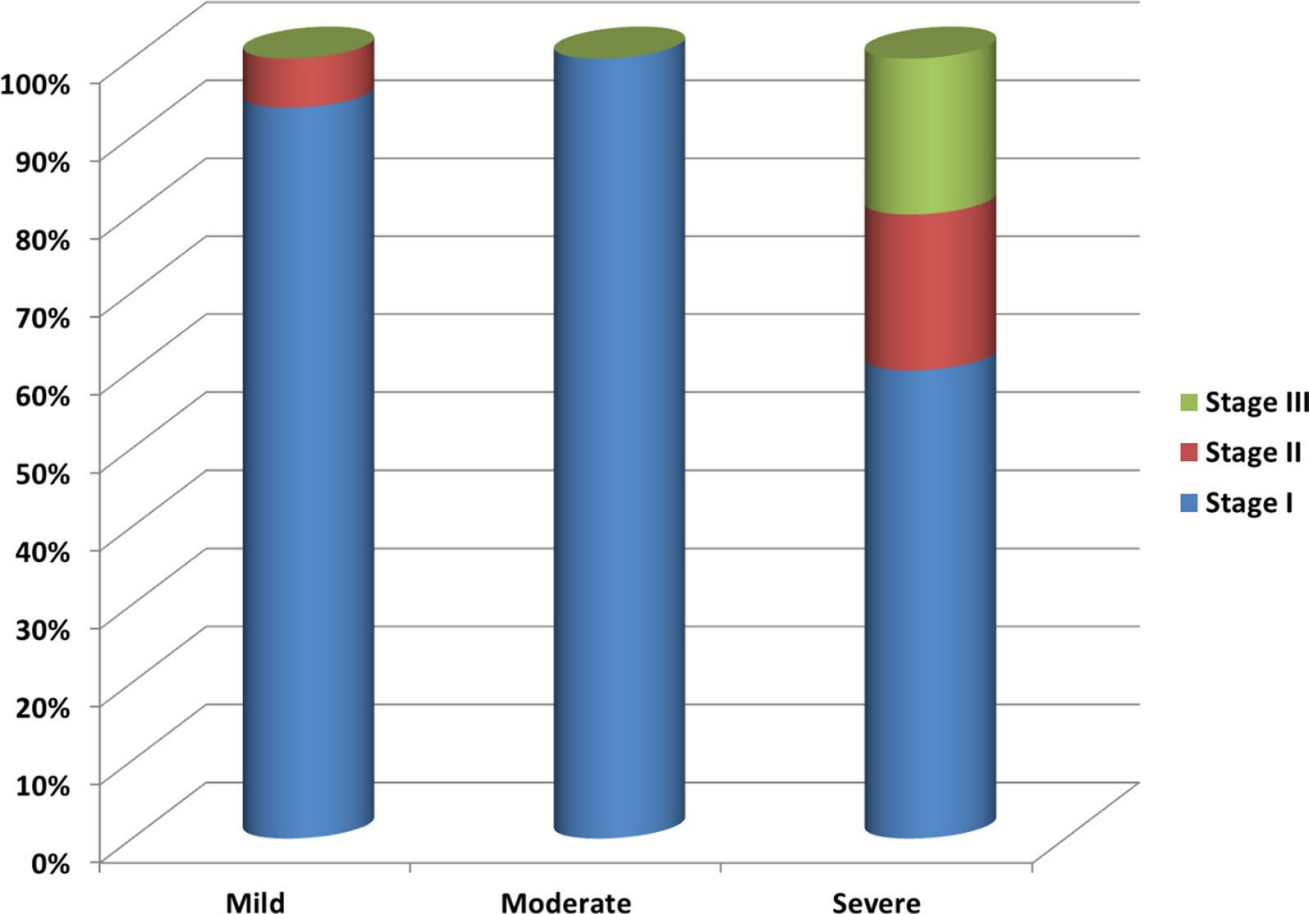
Comparing the tumor staging to average scores

The Correlation between the average scores and sites of tumor is shown in Fig. 2. The relation between the stage of the tumor and the average score is shown in Fig. 3. The relation between the type of oncoplastic technique and the average score is shown in Tables 4 and 5.

**Table 4:** Comparing the oncoplastic technique done against aesthetic score

Operation done	Average score					
	Mild (0–25)		Moderate (26–43)		Severe (≥ 44)	
	No	%	No	%	No	%
LS + NAC transfer + AC	46	75%	1	1.25%	3	3.75%
LS + NAC transfer + SLNB	2	2.5%	1	1.25%	1	1.25%
NSM + AC + ILDF	10	12.5%	2	2.5%	2	2.5%
SSM + AC + ILDMF	2	2.5%	4	5%	2	2.5%
Reduction mammoplasty + AC	4	5%	0	0%	0	0%

Lateral segmentectomy (LS); nipple-areola complex (NAC; sentinel lymph node biopsy (SLNB); axillary clearance (AC); nipple-sparing mastectomy (NSM); immediate latissimus dorsi myocutaneous flap (ILDMF); skin-sparing mastectomy (SSM)

**Table 5:** Correlation of different surgical techniques with aesthetic outcomes

The resection volume	OBS surgical techniques	Average score no (%)		
		Mild	Moderate	Severe
Type I	LS + NAC transfer + SLNB ± AC	48(77.5)	2(2.5)	4(5)
Type II	Reduction mammoplasty + AC	4(5)	0	0
Type III	SSM ± NSM + AC + ILDMF	12(15)	6(7.5)	4(5)

Type I: 20% of breast tissue removed, type II: 20–50% of breast tissue removed, type III:50% of breast tissue removed, Lateral segmentectomy (LS); nipple -areola complex (NAC; sentinel lymph node biopsy (SLNB); axillary clearance (AC); nipple-sparing mastectomy (NSM); immediate latissimus dorsi myocutaneous flap (ILDMF); skin-sparing mastectomy (SSM)

### Recurrent cases

#### Case 1

A 36-year-old female having a mass of 25 × 35 mm in the left breast associated with 2 hard palpable LNs > 20 mm presented at 21st month. T3 N2 M0 (stage III). Tru-cut needle tissue biopsy (US-guided) revealed G III infiltrating ductal carcinoma. Abdominal US, CXR, and PET scans were free. An elevation of CA15-3 and CEA were found. She underwent NSM + AC + ILDMF. Histopathological examination revealed grade II infiltrating ductal carcinoma, HER2 new, and PR were negative, but ER was positive.

#### Case 2

During clinical follow-up at 23rd month, a right breast mass of 15 × 20 mm was discovered. MRI spectroscopy of the breast showed a malignant mass of 18 × 19 mm present in the upper outer quadrant of the right breast. Core tissue was obtained by a Tru-cut needle, and GII infiltrating ductal carcinoma was detected. Abdominal us, CXR, and PET scans were free, and CA15-3 and CEA levels were high, indicating the probability of recurrence. She underwent SSM + AC + ILDMF. Postoperative pathology confirmed the same diagnosis. Modified radical mastectomy was done for the two cases of recurrence.

## Discussion

Oncoplastic techniques are a valid standard alternative to BCS for breast cancer patients, with little differences in complications and similar outcomes in reference to the number of local recurrence and metastases. Aesthetic outcomes after OBS and BCS are also considered important, along with the oncologic outcome, which is the primary goal of therapy [21, 22].

In this study, we focused on the aesthetic outcome along with other parameters of oncological safety in breast cancer patients undergoing OBS. Preoperative elevation of CEA and CA 15-3 levels has a direct relation to tumor burden, and both are independent prognostic factors for breast cancer [23]. This study showed that CEA was elevated in 60 cases (75%), while CA15-3 was elevated in 44 patients (55%).

In the current research, the rate of recurrence was calculated to be 2.5% during the variable periods of clinical follow-up (24–48 months). Most of the available studies have reported different recurrence rates based on the stage and size of tumors; therefore, it was difficult to corroborate our data with them [18, 20, 24]. Similar oncological results for both groups, OBS has also been shown [20].

OBS was similar with regards to tumor stage, histopathological type, tumor grading, intra-ductal component presence, hormonal receptor status, and nodal involvement. This suggested that local recurrences had no significant association with histopathological grading. Noticeably, we found that cases undergoing OBS had larger tumors. This suggested that the increased rate of local recurrences in OBS cases could be due to large tumor sizes as reported earlier [13].

Cases of the OBS group had larger tumors with lympho-vascular invasion. Unfortunately, we could not study the correlation between lympho-vascular invasion and local recurrence because of insufficient data. Removal of the primary growth with clear negative surgical margins is also an important factor for recurrence. In this study, all cases had negative surgical margins, but we observed recurrence in two cases.

Long-term outcomes of oncoplastic surgery are comparable or superior to standard BCS. Oncoplastic resections resulted in fewer positive margins (12 versus 21%) and fewer re-excisions (4 versus 15%), but a higher rate of completion mastectomy (7 versus 4%) compared with standard BCS [25].

At 3–5 years, patients who underwent oncoplastic resections developed fewer complications (16 versus 26%) and local recurrences (4 versus 7%) and had higher satisfaction with the appearance of their breast (90 versus 83%) [26].

Several reports note the safety of oncoplastic procedures with high rates of overall and disease-free survival and low rates of local recurrence, distant recurrence, re-excision, conversion to mastectomy, and complications [25–28].

The cosmetic appearance after mastectomy is just as important as after BCS. Several OBS techniques may enhance the aesthetic result of mastectomy since most women will live long lives after the initial treatment [2]. Body change stress is subjective psychological stress associated with surgeries of breast cancer. It is presented with traumatic stress-like symptoms in women with breast cancer and those undergoing surgery for it. An early psychometric foundation used the BITS to assess common and distressing Quality of Life (QoL) in patients with breast cancer [16, 29]. Body image and its assessment are the rising concerns of psychosocial oncology. Breast cancer management needs to manage traumatic stress in patients regarding their body image [30, 31].

BID is the intrusive thoughts and avoidant behaviors reflecting this stressor. BITS was constructed as a valid and reliable tool to assess BID and further reduce or prevent it [16, 32]. The aesthetic outcome, for all the patients, could be assessed in the patients by themselves or by means of photographs. Subjective visual assessment of cases by observers was, therefore, an integral part of BITS [29, 33].

Integrating OBS with other breast cancer treatments (Radiation treatment and neoadjuvant systemic therapy) achieves the comprehensive goal of aesthetic breast cancer therapy. Working with the radiation oncologist to properly target the right amount of radiation to the specific sites will contribute to improving the final look of the breast [4].

The total score of BITS ranges from 0 to 75 as it includes 15-item questionnaires. The severity of BID was determined by this score in the study. The average scores were recorded from the mean of the summation of scores in the 6–8 settings of follow-up for each woman. If the average score was presented against the side, stages of the tumor, site, hormonal receptor status, and the axillary LNs commitment, it was insignificant.

Although whole breast irradiation is a mandatory treatment for breast cancer after BCS and OBS, it may negatively impact the cosmetic result of the treated breast, thereby reducing the quality of life [34, 35]. The results of our study are coping with the data of these studies as the local effects of radiotherapy had a significant impact on the BITS scores and patient satisfaction.

Body image distress (BID) has been conceptualized as the perception and behavior of a person who had undergone breast cancer surgery. These cognitions and behavior usually have an anxiety-like responses. Breast impact of treatment scale (BITS) was developed to assess the intrusive and avoidant response of the patient to hypothesized traumatic events of breast cancer surgery. These intrusive response questions are for evaluation of pervasive thoughts and adoptive responses for cognitive experience [15, 16].

In this current research, most women (72 cases; 90%) were satisfied with the aesthetic outcomes from breast conservation treatment. About 10% (8 cases) of our patients were unsatisfied with their cosmetic outcome after undergoing OBS. This was higher than in another study, which reported 75% of patients satisfied with their aesthetic outcome at 48 months [36].

Detection of cases with sub-optimal aesthetic results would be more valuable after identification of statistically significant variables associated with woman dissatisfaction. The patient’s age was a significant variable. The mean age of satisfied patients was 61.2 years, while the mean age of unsatisfied patients was 52.3 years. This indicates a greater risk of poor cosmetic outcomes could be detected with younger patients, as demonstrated in other studies [36].

Our results are coping with the previous cumulative data indicating that OBS provides tumor resection with larger resection margins and acceptable oncologic and cosmetic outcomes. OBS can achieve higher rates of negative margins with low recurrence and better cosmetics. OBS has a positive impact on the QoL and the self-esteem of patients [27, 28, 37].

We recommend oncoplastic options to be considered for every breast cancer to ensure comprehensive treatment results in optimal survivorship, encompassing oncologic, functional, and aesthetic outcomes. The merit of this study was that all women were evaluated regularly over a relatively long duration (24–48 months) by their physicians. So, it can add to the field of monitoring of aesthetic outcomes in patients of OBS.

Our study had some limitations such as the lack of randomization and the disparity in skills between facilities and between surgeons, which could cause selection bias. The sample size of the study was a significant limitation as larger multicenter samples could allow more critical analysis. The BITS score is not a specific tool to evaluate aesthetic outcomes and we should consider other aesthetic evaluation tools to assess and validate a more appropriate questionnaire for continuing this study.

## Conclusion

The oncoplastic breast surgeries are feasible and have a high rate of oncological safety with only a 2.5% of recurrence rate in conjunction with the maintenance of good aesthetic outcome as most of the patients were satisfied with the aesthetic outcome having mild BID and mild BITS of OBS. In future comparative effectiveness studies and outcome reporting, it might be useful to measure cosmetic outcomes using these measures.

## Data Availability

The datasets used and/or analyzed during the current study are available from the corresponding author on reasonable request.

## Abbreviations

OBS: Oncoplastic Breast surgeries
LS: Lateral segmentectomy
NAC: Nipple-areola complex
SLNB: Sentinel lymph node biopsy
AC: Axillary clearance
NSM: Nipple-sparing mastectomy
ILDMF: Immediate latissimus dorsi myocutaneous flap
SSM: Skin-sparing mastectomy
BID: Body image distress
BITS: Breast impact of treatment scale
QoL: Quality of Life

## References

[1] Sherwell-Cabello S, Maffuz-Aziz A, Villegas-Carlos F, Domínguez-Reyes C, Labastida-Almendaro S, Rodríguez-Cuevas S (2015). Feasibility, and cosmetic outcome of oncoplastic surgery in breast cancer treatment. Cirugía y Cirujanos.

[2] Pesce CE, Liederbach E, Czechura T, Winchester DJ, Yao K (2014). Changing surgical trends in young patients with early-stage breast cancer, 2003 to 2010: a report from the National Cancer Data Base. J Am Coll Surg.

[3] Association of Breast Surgery at BASO, Association of Breast Surgery at BAPRAS, Training Interface Group in Breast Surgery, Baildam A, Bishop H, et al. Oncoplastic breast surgery—a guide to good practice. Eur J Surg Oncol. 2007;33(Suppl 1):S1–23. 10.1016/j.ejso.2007.04.014 17604938

[4] Clough KB, Kaufman GJ, Nos C, Buccimazza I, Sarfati IM (2010). Improving breast cancer surgery: a classification and quadrant per quadrant atlas for oncoplastic surgery. Ann Surg Oncol.

[5] Veiga DF, Veiga-Filho J, Ribeiro LM, Archangelo-Junior I, Mendes DA, Andrade VO (2011). Evaluations of aesthetic outcomes of oncoplastic surgery by surgeons of different gender and specialty: a prospective controlled study. Breast.

[6] Early Breast Cancer Trialists' Collaborative Group (EBCTCG). Long-term outcomes for neoadjuvant versus adjuvant chemotherapy in early breast cancer: meta-analysis of individual patient data from ten randomised trials. Lancet Oncol. 2018;19(1):27–39. 10.1016/S1470-2045(17)30777-5 PMC5757427 29242041

[7] Losken A, Hamdi M (2009). Partial breast reconstruction: current perspectives. Plast Reconstr Surg.

[8] Haloua MH, Krekel NM, Winters HA, Rietveld DH, Meijer S, Bloemers FW (2013). A systematic review of oncoplastic breast-conserving surgery: current weaknesses and prospects. Ann Surg.

[9] Jeevan R, Cromwell DA, Trivella M, Lawrence G, Kearins O, Pereira J, et al. Reoperation rates after breast conserving surgery for breast cancer among women in England: retrospective study of hospital episode statistics. BMJ. 2012;345:e4505. 10.1136/bmj.e4505 PMC3395735 22791786

[10] Morrow M, Van Zee KJ, Solin LJ, Houssami N, Chavez-MacGregor M, Harris JR (2016). Society of Surgical Oncology-American Society for Radiation Oncology-American Society of Clinical Oncology consensus guideline on margins for breast-conserving surgery with whole-breast irradiation in ductal carcinoma in situ. Pract Radiat Oncol.

[11] Houssami N, Macaskill P, Marinovich ML, Dixon JM, Irwig L, Brennan ME (2010). Meta-analysis of the impact of surgical margins on local recurrence in women with early-stage invasive breast cancer treated with breast-conserving therapy. Eur J Cancer.

[12] Carter SA, Lyons GR, Kuerer HM, Bassett RL, Oates S, Thompson A (2016). Operative and oncologic outcomes in 9861 patients with operable breast cancer: single-institution analysis of breast conservation with oncoplastic reconstruction. Ann Surg Oncol.

[13] Chakravorty A, Shrestha AK, Sanmugalingam N, Rapisarda F, Roche N, Querci Della Rovere G, et al. How safe is oncoplastic breast conservation? Comparative analysis with standard breast conserving surgery. Eur J Surg Oncol. 2012;38(5):395–8. 10.1016/j.ejso.2012.02.186 22436560

[14] De La Cruz L, Blankenship SA, Chatterjee A, Geha R, Nocera N, Czerniecki BJ (2016). Outcomes after oncoplastic breast-conserving surgery in breast cancer patients: a systematic literature review. Ann Surg Oncol.

[15] White CA (2000). Body image dimensions and cancer: a heuristic cognitive behavioural model. Psychooncology.

[16] Frierson GM, Thiel DL, Andersen BL (2006). Body change stress for women with breast cancer: the breast-impact of treatment scale. Ann Behav Med.

[17] Chan SWW, Chueng PSY, Lam SH (2010). Cosmetic outcome and percentage of breast volume excision in oncoplastic breast conserving surgery. World J Surg.

[18] Clough KB, Lewis JS, Couturaud B, Fitoussi A, Nos C, Falcou M-C (2003). Oncoplastic techniques allow extensive resections for breast-conserving therapy of breast carcinomas. Ann Surg.

[19] Meretoja TJ, Svarvar C, Jahkola TA (2010). Outcome of oncoplastic breast surgery in 90 prospective patients. Am J Surg.

[20] Rietjens M, Urban CA, Rey PC, Mazzarol G, Maisonneuve P, Garusi C (2007). Long-term oncological results of breast conservative treatment with oncoplastic surgery. Breast.

[21] Petit JY, De Lorenzi F, Rietjens M, Intra M, Martella S, Garusi C (2007). Technical tricks to improve the cosmetic results of breast-conserving treatment. Breast.

[22] Calì Cassi L, Vanni G, Petrella G, Orsaria P, Pistolese C, Lo Russo G (2016). Comparative study of oncoplastic versus non-oncoplastic breast conserving surgery in a group of 211 breast cancer patients. Eur Rev Med Pharmacol Sci.

[23] Park BW, Oh JW, Kim JH, Park SH, Kim KS, Kim JH (2008). Preoperative CA 15-3 and CEA serum levels as predictor for breast cancer outcomes. Ann Oncol.

[24] Ueda S, Tamaki Y, Yano K, Okishiro N, Yanagisawa T, Imasato M (2008). Cosmetic outcome and patient satisfaction after skin-sparing mastectomy for breast cancer with immediate reconstruction of the breast. Surgery.

[25] Fitoussi AD, Berry MG, Famà F, Falcou MC, Curnier A, Couturaud B (2010). Oncoplastic breast surgery for cancer: analysis of 540 consecutive cases [outcomes article]. Plast Reconstr Surg.

[26] Losken A, Dugal CS, Styblo TM, Carlson GW (2014). A meta-analysis comparing breast conservation therapy alone to the oncoplastic technique. Ann Plast Surg.

[27] Urban C, Lima R, Schunemann E, Spautz C, Rabinovich I, Anselmi K (2011). Oncoplastic principles in breast conserving surgery. Breast.

[28] Patani N, Carpenter R (2010). Oncological and aesthetic considerations of conservational surgery for multifocal/multicentric breast cancer. Breast J.

[29] Cardoso MJ, Cardoso JS, Wild T, Krois W, Fitzal F (2009). Comparing two objective methods for the aesthetic evaluation of breast cancer conservative treatment. Breast Cancer Res Treat.

[30] Fayman MS, Potgieter E, Becker PJ (2006). The pedicle tram flap: a focus on improved aesthetic outcome. Aesthet Plast Surg.

[31] Fitzal F, Krois W, Trischler H, Wutzel L, Riedl O, Kühbelböck U (2007). The use of a breast symmetry index for objective evaluation of breast cosmesis. Breast.

[32] Collins KK, Liu Y, Schootman M, Aft R, Yan Y, Dean G (2011). Effects of breast cancer surgery and surgical side effects on body image over time. Breast Cancer Res Treat.

[33] Reefy S, Patani N, Anderson A, Burgoyne G, Osman H, Mokbel K (2010). Oncological outcome and patient satisfaction with skin-sparing mastectomy and immediate breast reconstruction: a prospective observational study. BMC Cancer.

[34] Early Breast Cancer Trialists’ Collaborative G, Darby S, McGale P. Effect of radiotherapy after breast-conserving surgery on 10-year recurrence and 15-year breast cancer death: meta-analysis of individual patient data for 10,801 women in 17 randomised trials. Lancet. 2011;378:1707–16. 10.1016/S0140-6736(11)61629-2 PMC3254252 22019144

[35] Hahn EA, Segawa E, Kaiser K. Health-related quality of life among women with ductal carcinoma in situ or early invasive breast cancer: validation of the fact-b (version 4) Expert Review of Quality of Life in Cancer Care. 2016;1:99–109.

[36] Beadle GF, Silver B, Botnick L, Hellman S, Harris JR (1984). Cosmetic results following primary radiation therapy for early breast cancer. Cancer.

[37] Nair A, Jaleel S, Abbott N, Buxton P, Matey P (2010). Skin-reducing mastectomy with immediate implant reconstruction as an indispensable tool in the provision of oncoplastic breast services. Ann Surg Oncol.

